# Engineering nanosystems for regulating reproductive health in women

**DOI:** 10.7150/thno.102626

**Published:** 2025-01-01

**Authors:** Qinrui Fu, Lejun Fu

**Affiliations:** 1 Key Laboratory of Birth Regulation and Control Technology of National Health Commission of China, Maternal and Child Health Care Hospital of Shandong Province Affiliated to Qingdao University, Qingdao University, Jinan 250014, China.; 2 Institute for Translational Medicine, College of Medicine, Qingdao University, Qingdao 266021, China.; 3 School of Chemistry and Materials Science, Anhui Normal University, Wuhu 230022, China.

**Keywords:** nanomedicine, woman's reproductive health, diagnosis, treatment, reproductive health regulation

## Abstract

Reproductive health-related diseases have a significant impact on the well-being of millions of women worldwide, severely compromising their quality of life. Women encounter unique challenges in terms of reproductive health, including gynecological diseases and malignant neoplasms prior to pregnancy, as well as complications during pregnancy that greatly undermine their physical and mental health. Despite recent advancements in the field of female reproduction, substantial challenges still persist. To address these challenges, nanotechnology-based diagnostic and therapeutic strategies have emerged to provide intelligent detection and treatment for pathologies related to women's reproductive health. Although some progress has been made with nanotherapeutics in this domain, its application is still nascent due to the delicate and intricate nature of the female reproductive system. This review comprehensively presents the latest advancements in nanomedicine for regulating woman's reproductive health. Firstly, based on the time period of onset, nanomedicine applications are categorized into four subcategories: 1) preconception diseases such as polycystic ovary syndrome, endometriosis, and gynecologic malignancy treatment; 2) pregastrulation period diseases including placenta accreta spectrum disorders and ectopic pregnancy; 3) mid-term pregnancy diseases like preeclampsia; and 4) late pregnancy diseases such as deep vein thrombosis during pregnancy. The systematic introduction covers the progress made by nanomedicine in various disease areas. Finally, this article discusses the challenges faced by these nanomedicines from research to clinical translation while also highlighting future directions.

## Introduction

The significance of reproductive health for women is self-evident. Specifically, the unique physiological structure inherent to women necessitates a delicate reproductive health environment 1. Inadvertently contracting various bacterial inflammations poses a threat to fertility and both physical and mental well-being, thereby significantly impacting their overall quality of life 2,3.

The detrimental impact on female reproductive health is evident in at least two dimensions: firstly, physical well-being; the interdependence between the health of the female reproductive system and pregnancy is profound. Any malfunction within any component of the female reproductive system can result in infertility or have adverse effects on fetal development. In particular, pregnancy during acute inflammation may lead to miscarriage or hinder proper fetal growth. Secondly, mental well-being; issues concerning female reproductive health are often accompanied by complications that induce psychological distress among women, potentially leading to diminished self-esteem, social withdrawal, and other psychological ailments 4.

The medical field of “Women's reproductive health” encompasses the diagnosis and treatment of diseases and disorders that impact women's reproductive well-being 5. Women encounter distinctive health challenges, including complications during pregnancy 6,7, gynecological diseases 8,9, and malignancies 10,11. In comparison to men, women exhibit a heightened susceptibility to conditions such as breast cancer 12,13, urinary incontinence 14,15, and thrombus due to their unique biological characteristics. Due to the unique characteristics of female reproductive organs, many women experience pain and anxiety regarding diagnosis and treatment, leading to their reluctance in participating in regular examinations and accepting routine diagnosis and treatment. Consequently, this results in the progression of treatable diseases into fatal conditions. Therefore, it is imperative to meticulously design treatment approaches and diagnostic strategies tailored specifically towards addressing the multifaceted aspects surrounding women's health.

Rapid advancements in nanotechnology offer a promising strategy for the development of effective and personalized treatments across various diseases 16-20, with the potential to revolutionize women's health in multiple key domains 21-23. Nanomedicine holds extensive application prospects within women's reproductive health 24-28. By designing responsive or targeted nanosystems 29-31, therapeutic drugs can be accurately delivered to specific regions of the reproductive system 32,33, offering novel treatment options for infertility, polycystic ovary syndrome, and other ailments 5. For instance, targeted drug delivery systems can be developed to enhance efficacy while minimizing side effects, thereby aiding in the treatment of gynecological cancer and endometriosis 34. Additionally, precise and real-time detection of gynecological diseases can be achieved through the design of imaging contrast agents 35. Nanomedicine also exhibits significant potential in addressing pregnancy complications and improving maternal as well as child health. Smart nanomedicines could be designed to enable specific delivery of therapeutic drugs or contrast agents to the placenta or fetus—an essential aspect for diagnosing and treating conditions like ectopic pregnancy, pre-eclampsia, or fetal growth restriction.

**Figure 1:**
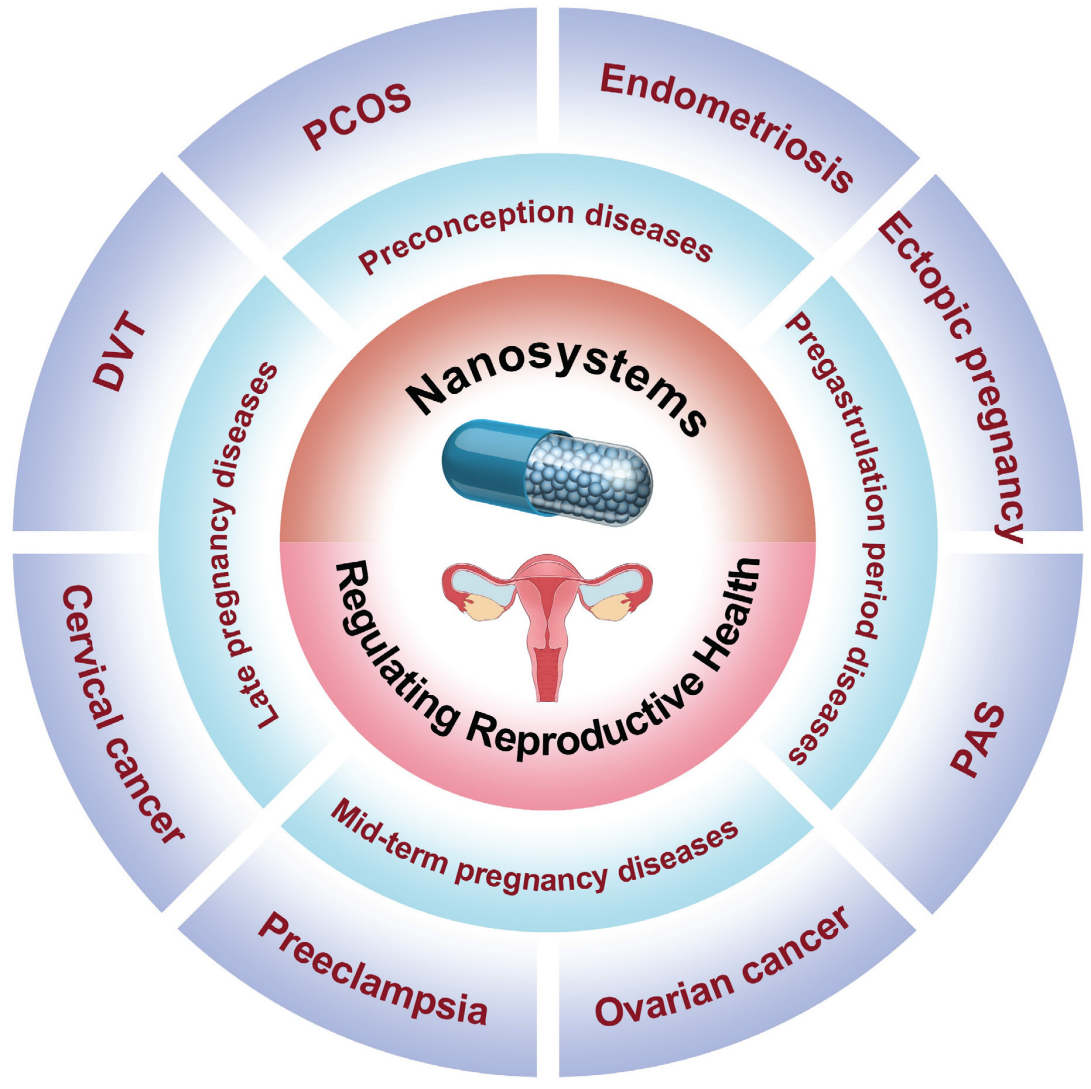
Schematic illustration of engineering nanosystems for regulating women's reproductive health (PCOS: polycystic ovary syndrome; PAS: placental accreta spectrum; DVT: deep vein thrombosis).

To our knowledge, there is a scarcity of comprehensive reviews summarizing advancements in female reproductive regulation using nanomedicine, despite the numerous studies conducted on nanomedicine in disease diagnosis and treatment. In this review, we provide a comprehensive overview of recent applications of nanomedicine in women's reproductive regulation, as depicted in** Figure 1**. The Review categorizes the application of nanomedicine in reproductive regulation into four groups based on women's physiological characteristics and different stages of diseases. These groups include: 1) preconception diseases, such as polycystic ovary syndrome, endometriosis, and gynecologic malignancy treatment; 2) pregastrulation period diseases, including placenta accreta spectrum disorders and ectopic pregnancy; 3) mid-term pregnancy diseases, such as preeclampsia; and 4) late pregnancy diseases, like deep vein thrombosis during pregnancy and prenatal intervention for fetal health. Additionally, we explore the prospects and potential opportunities for utilizing nanomedicine in diagnosing and treating fertility-related disorders in women, offering valuable insights into future developments involving nanomaterials.

## Preconception diseases

### Polycystic ovary syndrome

Polycystic ovary syndrome (PCOS), affecting 5-20% of women of reproductive age globally, represents the most prevalent endocrine disorder among females and is characterized by hirsutism, anovulation, and polycystic ovaries 36-39. Furthermore, PCOS can lead to anovulatory infertility and early-onset type II diabetes in women 40; hence, it is imperative to develop targeted approaches for diagnosis and treatment 41.

The standard drug previously introduced for the treatment of PCOS was clomiphene; however, a third of the patients indicated resistance to this medication. Therefore, there is a need for new combinations of drugs or supplements to address these specific patient groups. Nanomedicine holds immense potential in enhancing current diagnostic and therapeutic strategies for PCOS. Nanomaterials with diverse compositions have long been acclaimed for their ability to encapsulate hydrophilic and hydrophobic molecules, including small molecule drugs, imaging contrast agents, and nucleic acids. They not only protect these cargoes from degradation but also prolong their circulation time within the body while delivering higher quantities to targeted tissues of interest. By functionalizing nanoparticle surfaces with active targeting moieties, even greater accumulation of encapsulated payloads can be achieved in specific tissues while minimizing accumulation in non-targeted sites. This approach helps reduce the severity of systemic side effects associated with various drugs.

#### Application of nanomaterials for diagnosis of PCOS

PCOS, one of the most prevalent gynecological disorders, results in hormonal dysregulation closely associated with ovarian function, including luteinizing hormone (LH) and follicle-stimulating hormone (FSH), among others 42. Research has demonstrated that elevated LH levels, reduced FSH levels, and an increased LH/FSH ratio serve as crucial diagnostic indicators for PCOS 43,44. For instance, Chauhan *et al*. developed a novel electrochemical sensor based on nano-molecular imprinting polymer technology 45. This sensor utilizes electropolymerization to selectively and specifically detect FSH by forming a molecularly imprinted polymer film onto a nanomaterials-modified indium tin oxide (ITO) electrode composed of NiCo_2_O_4_/rGO for accurate FSH detection in individuals with PCOS.

**Figure 2:**
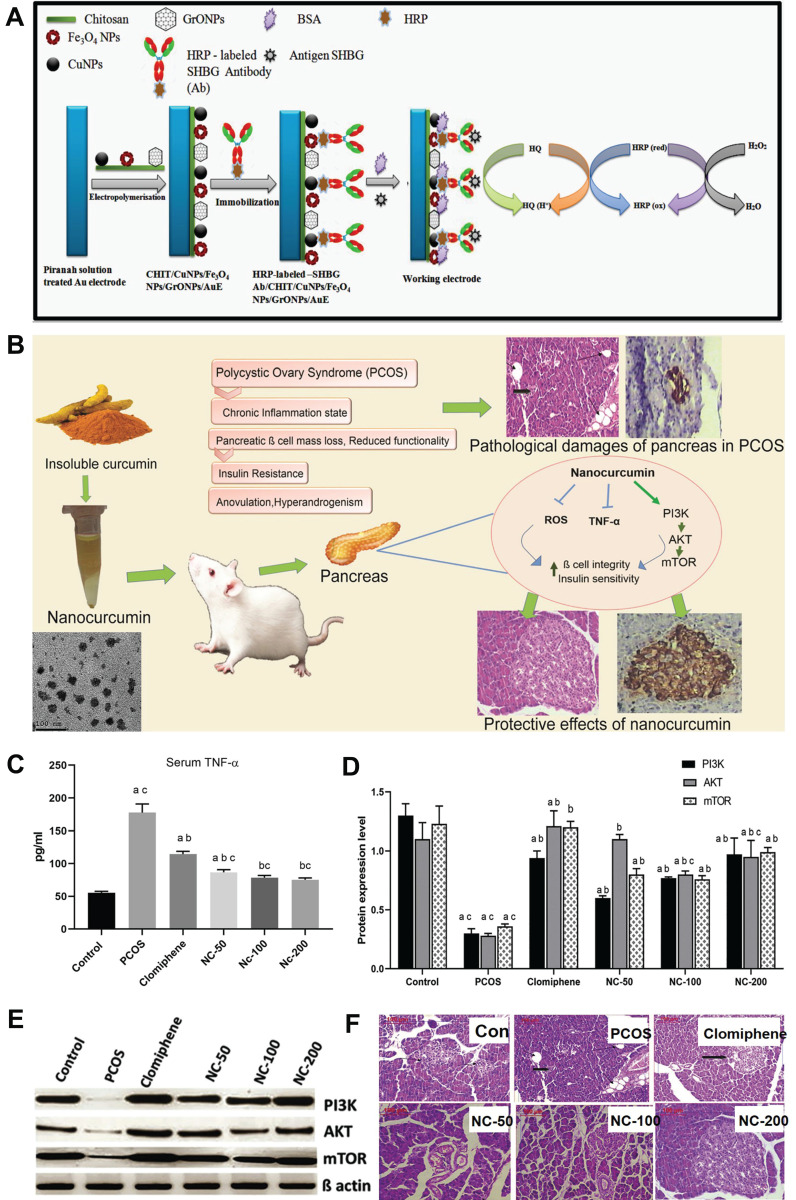
(A) Schematic representation illustrating the chemical reactions involved in the synthesis of HRP-SHBG Ab/CHIT/CuNPs/Fe_3_O_4_NPs/GrONPs/AuE. (B) TEM image of nanocurcumin. (C) Changes of serum TNF-α levels after different treatments. (D) The expression levels of PI3K, AKT and mTOR proteins after different treatments. (E) PI3K, AKT and mTOR western blotting after different treatments. (F) Histomorphology of pancreatic tissue after different treatments. **(A)** Adapted with permission 49, copyright 2019, Elsevier Inc. **(B-F)** Adapted with permission 57, copyright 2020, Elsevier Inc.

Elevated androgen levels are a hallmark symptom of PCOS patients, serving as the primary driving factor behind impaired follicular development, abnormal ovulation, and endometrial lesions in these individuals. Consequently, assessing androgen levels is crucial for diagnosing PCOS. Sex hormone-binding globulin (SHBG), an essential carrier protein regulating androgen activity, can be utilized to diagnose PCOS due to its identification as a biomarker for this condition 46. Therefore, it is crucial to study and analyze SHBG levels in order to accurately diagnose PCOS patients 47,48. For instance, Pundir *et al*. developed an innovative electrochemical immunosensor utilizing a gold electrode modified with chitosan (CHIT), copper nanoparticles (CuNPs), iron nanoparticles (Fe_3_O_4_NPs), and graphene oxide nanoparticles (GrONPs) 49. This sensor incorporates horseradish peroxidase-labeled SHBG antibody immobilized onto the CHIT/CuNPs/Fe_3_O_4_ NPs/GrONPs/Au surface, allowing for specific detection of SHBG antigen in PCOS patients with a lower limit of detection (LOD) at 0.01 nM (**Figure 2A**).

Nanotechnology offers significant potential for the detection and evaluation of human reproductive hormone proteins associated with PCOS, such as LH, FSH, prolactin (PRL), and anti-Müllerian hormone (AMH). Li *et al*. reported a quadruplex ultrasensitive immunoassay capable of simultaneously assessing these four hormone proteins in biofluid samples 50. This innovative assay employs single-molecule imaging of microwell arrays and capture antibody nanobeads to develop multiplex bead array immunoassays. The analyte-bound beads are easily segregated into individual wells and detected using fluorophores that emit distinct wavelengths corresponding to the nanobeads. The proposed quadruplex immunoassay demonstrates excellent 4-parameter logistic calibration curves, with ranges spanning from 2.7 to 2000 pg/mL for FSH, 1.6 to 1200 pg/mL for LH, 1.8 to 1300 pg/mL for PRL, and 0.3 to 220 pg/mL for AMH. The limits of detection are impressively low, measured at 0.32 pg/mL for FSH, 0.28 pg/mL for LH, 0.14 pg/mL for PRL, and an exceptional 0.02 pg/mL for AMH. Furthermore, the developed quadruplex immunoassay was tested on clinical venous serum samples, where it exhibited remarkable consistency with clinical test results, thereby demonstrating its efficacy in diagnosing PCOS.

#### Application of nanomaterials in the treatment of PCOS

The intricate pathology of PCOS represents a significant health and socio-economic burden. Recent investigations have explored the potential therapeutic or regulatory effects of botanical agents, including Trigonella foenum-graecum L 51,52, camellia sinensis 53,54, cinnamon 55, curcumin 56, and aloe vera in managing PCOS. Perturbations in the PI3K/AKT/mTOR signaling pathways and elevated levels of tumor necrosis factor-α (TNF-α) have been implicated in the pathogenesis of PCOS.

Morcos* et al*. developed and synthesized a nanocurcumin formulation with an average particle size of 30±7 nm to investigate its therapeutic potential in letrozole-induced PCOS-related pancreatic deficiencies by regulating the PI3K/AKT/mTOR pathway and TNF-α levels, which are irregularities observed in β cells of the PCOS rat model (**Figure 2B**) 57. Compared to free curcumin, nanocurcumin exhibits enhanced water solubility and bioavailability, thereby significantly increasing its therapeutic potential in PCOS-related pancreatic deficiencies. The letrozole-induced PCOS group exhibited PCOS symptoms such as disturbed androgens, dyslipidemia, and ovarian histopathological changes; however, after treatment with NC-100 (oral nanocurcumin solution ingestion at a dose of 100 mg/kg body weight) and NC-200 (oral nanocurcumin solution ingestion at a dose of 200 mg/kg body weight), serum samples were obtained to measure sex hormone levels. The experimental results demonstrated that nanocurcumin effectively restored serum sex hormones to their normal ranges in a dose-dependent manner. No significant differences were observed in progesterone, total cholesterol (TC), LDL-cholesterol (LDL-C), HDL-cholesterol (HDL-C), and insulin levels after nanocurcumin treatment compared to the control group. Furthermore, treatment with nanocurcumin restored oxidative stress markers malondialdehyde level (MDA), reduced glutathione (GSH), and superoxide dismutase enzyme activity back to normal levels in the treatment group. Additionally, an increase in nanocurcumin dosage effectively suppressed TNF-α expression according to serum inflammation standards shown in **Figure 2C**. **Figure 2D, E** revealed a significant reduction of PI3K, AKT, and mTOR protein expressions in the letrozole-induced PCOS group compared to the control group; however, nanocurcumin treatment successfully counteracted this disorder by restoring normal sex hormone levels. The histomorphology of pancreatic tissue revealed significant pathological impairments in the PCOS group, characterized by vacuolation and a marked decrease in β-cells. Treatment with nanocurcumin preserved the integrity of pancreatic tissue, with most Langerhans islets remaining intact (**Figure 2E**). These findings underscored the potential of nanocurcumin as a promising therapeutic approach for addressing pancreatic function deficits associated with PCOS, suggesting its broad applicability in treating this condition.

### Endometriosis

Endometriosis is a debilitating and systemic disorder characterized by the ectopic presence of uterine-like tissue 58-60. Epidemiological studies have reported that approximately 176 million women and girls worldwide are affected by this condition 61. Despite being a non-malignant gynecological disease, endometriosis can give rise to various deleterious manifestations including pelvic pain, dysmenorrhea, and infertility 62-65. However, despite significant advancements in medical research, an effective cure for this ailment remains elusive. Consequently, there is an imperative need for innovative therapeutic strategies to address the complexities associated with endometriosis.

**Figure 3:**
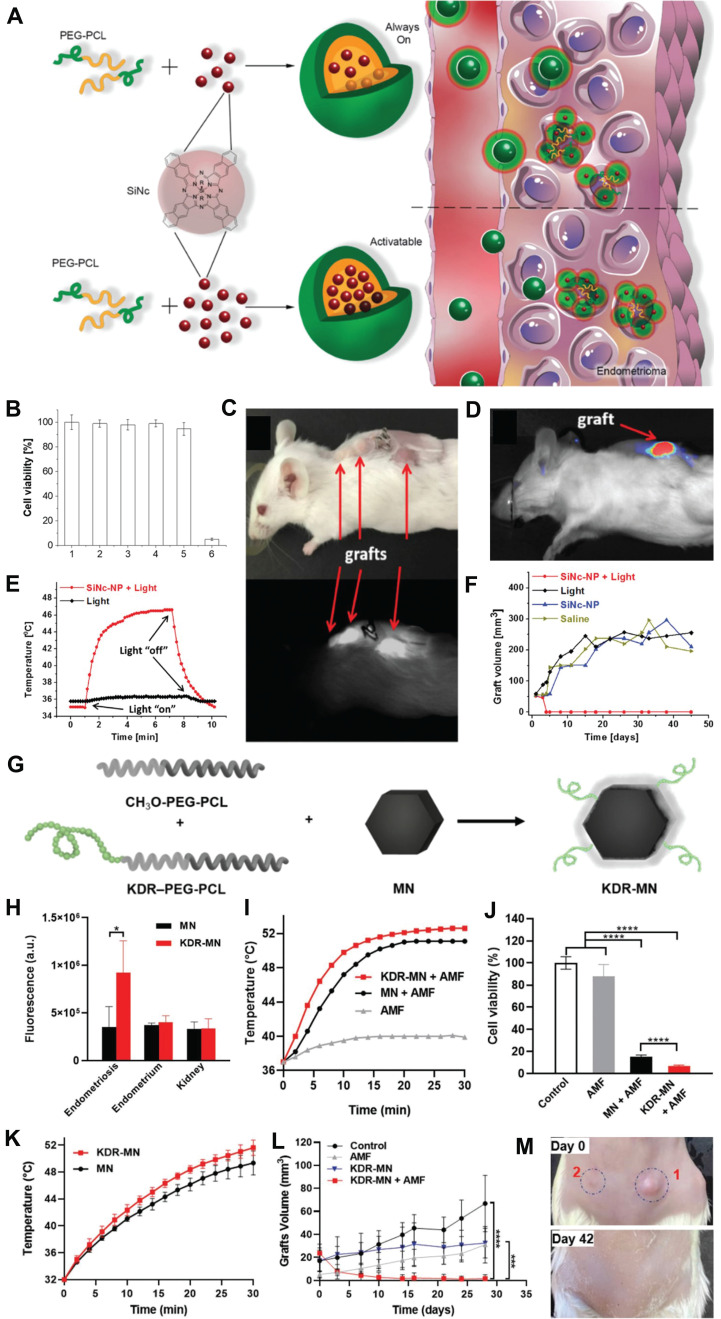
(A) Diagram of “normally open” and “activable” SiNc-loaded PEG-PCL nanoparticles (SiNc-NP). (B) Activity of stromal cells in macaque endometriosis after different treatments. (C) Mice in the endometriosis area 24 hours after intravenous injection of “activatable” SiNc-NP. (D) Near-infrared fluorescence images of mice carrying transplanted and resected tissue from endometriosis. (E) Temperature curves of plants with endometriosis displacement after different treatments. (F) The growth of subcutaneous endometriosis displacement plants after different treatments. (G) Diagram of KDR-MN synthesis. (H) Cell uptake of endometriosis stromal cells, endometrial cells and kidney cells by nanomaterials in rhesus monkeys after different treatments. (I) Temperature curves of endometriosis cells after different treatments. (J) The viability of endometriosis cells after different treatments. (K) After different treatments, endometriosis shifted the temperature curve inside the plant. (L) Growth curves of grafts after different treatments. (M) Photos of mice with endometriosis displacement plants before and after treatment. **(A-F)** Adapted with permission 66, copyright 2020, Wiley-VCH. **(G-M)** Adapted with permission 67, copyright 2022, Wiley-VCH.

For instance, Taratula* et al*. reported a nanoplatform composed of poly(ethylene glycol)-poly(caprolactone) (PEG-PCL) based polymer nanoparticles coated with silyl phthalocyanine (SiNc), which enables real-time near-infrared (NIR) fluorescence imaging and photothermal treatment of endometriosis (**Figure 3A**) 66. The *in vitro* experiments demonstrated that, following a 2-day incubation with endometrial stromal cells of macaque monkeys with endometriosis, SiNc-NPs exhibited remarkable efficacy in treatment by inducing more than 95% cell death in endometriotic cells upon exposure to 780 nm NIR light for 15 minutes (**Figure 3B**). The *in vivo* imaging experiments revealed that the administration of SiNC-NP *via* tail vein injection in mice with subcutaneous macaque endometrio-shifted implants resulted in its accumulation within endometriosis lesions. The NIR fluorescence of SiNC-NP was activated within 24 h following a single injection, enabling precise classification of the endometriotic lesions (**Figure 3C, D**). The *in vivo* treatment results demonstrated a rapid increase in temperature within endometriotic grafts, reaching up to 47 °C during photothermal therapy facilitated by intravenously administered SiNc-NP combined with 780 nm light (**Figure 3E**). Furthermore, complete eradication of endometriotic lesions was observed within 4 days following a single treatment of intravenous injection of SiNc-NPs into mice with an endometriosis model and subsequent NIR light irradiation for 15 minutes, without any recurrence throughout the entire duration of the 7-week study (**Figure 3F**).The potential acute toxicity of SiNc-NP on various organs was assessed by measuring surrogate biomarkers in the blood, including blood urea nitrogen and creatinine for kidney function, alkaline phosphatase and alanine aminotransferase for liver function, creatine kinase for muscle and heart function, as well as blood electrolytes and proteins as indicators of major organ toxicity. However, there were no statistically significant differences in these measurements between non-treated mice and mice injected with SiNc-NP, suggesting that the SiNc-NP do not exhibit any acute toxicity. These findings suggested that SiNc-NPs may serve as a promising and safe nanoplatform for the treatment of endometriosis.

In 2022, Taratula *et al*., developed a peptide-modified hexagonal iron oxide nanoparticle coated with PEG-PCL nanocarrier to specifically target vascular endothelial growth factor receptor 2 (VEGFR-2), also known as kinase insert domain receptor (KDR), aiming to enhance the therapeutic efficacy for endometriosis treatment (**Figure 3G**) 67. *In vitro* fluorescence imaging experiments demonstrated that the mean fluorescence intensity of endometriosis cells treated with KDR-targeting magnetic nanoparticles (MN) was 2.5-fold higher compared to unmodified nanoparticles (**Figure 3H**). The therapeutic effects of KDR-targeted MN (KDR-MN) were evaluated both *in vitro* and *in vivo*. Specifically, KDR-MN exhibited a faster increase in temperature above 46 °C compared to non-targeted MN, and this effect persisted for a longer duration, resulting in enhanced eradication of macaque endometriosis cells under an alternating magnetic field (AMF) (**Figures 3I, J**). In the* in vivo* experiments, mice with endometriosis models were intravenously injected with clinically relevant doses of KDR-MN. As depicted in **Figure 3L, M**, accumulation of KDR-MN within the proliferative graft led to an elevation in temperature under AMF and ultimately elimination of the proliferative lesion. These findings suggested that nanoparticle-mediated magnetothermal therapy may offer a promising non-surgical approach for eradicating endometriosis lesions and potentially revolutionize the treatment paradigm for this condition.

### Gynecologic malignancy treatment

#### Cervical cancer

**Figure 4:**
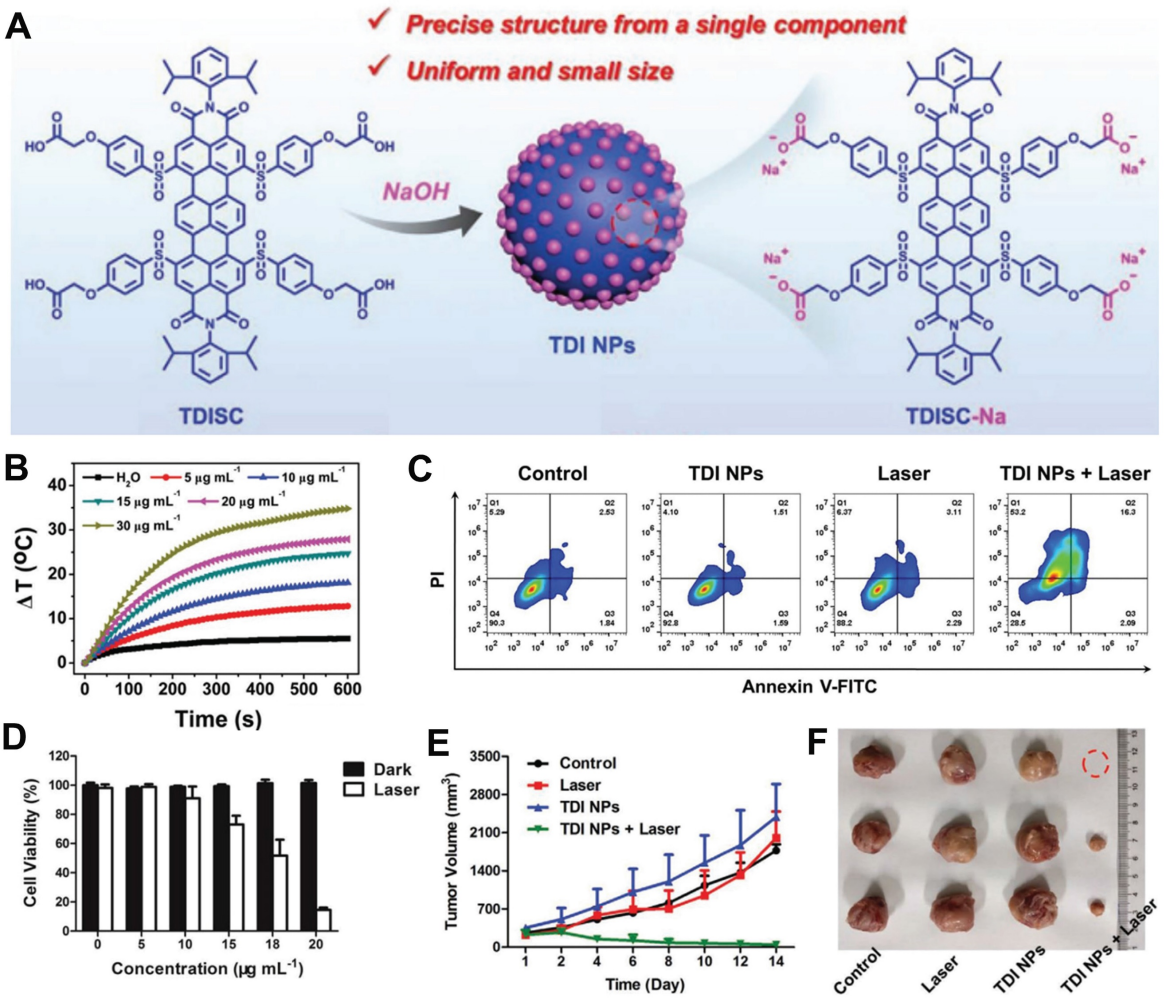
(A) Preparation of TDI NPs and its application in tumor. (B) Photothermal properties of TDI NPs at different concentrations after 685 nm laser irradiation. (C) Apoptosis analysis of HeLa cells incubated by TDI NPs after different treatments. (D) Photothermal effect of TDI NPs on HeLa. (E) Growth curves of tumor volume in mice after different treatments. (F) Representative pictures of removed tumors in mice on day 14 of treatment. **(A-F)** Adapted with permission 70, copyright 2021, Wiley-VCH.

Cervical cancer, with an estimated 604,000 new cases and 342,000 deaths in 2020, ranks as the fourth most prevalent cancer globally and remains one of the deadliest malignancies affecting women 68,69. Currently, there is a lack of effective treatment options for this disease. In light of this challenge, Sun *et al*. developed developed organic nanoparticles composed of terrylenediimide (TDI) NPs, derived from a TDI-based single component without the use of additives, for effective photothermal therapy in cervical cancer (**Figure 4A**) 70. Subsequently, the photothermal effects of TDI nanoparticles at various concentrations were investigated. As depicted in **Figure 4B**, higher nanoparticle concentrations exhibited rapid temperature elevation indicative of their potential therapeutic efficacy. Furthermore, *in vitro* experiments demonstrated that increasing nanoparticle concentration under laser irradiation significantly enhanced therapeutic effects on Hela cells, leading to a notable increase in apoptosis rate (**Figure 4C, D**). *In vivo* cell experiments further confirmed that TDI NPs combined with laser treatment effectively reduced tumor volume in mice and achieved favorable therapeutic outcomes (**Figures 4E, F**).

#### Ovarian cancer

Ovarian cancers stand as the foremost cause of fatalities stemming from gynecological malignancies worldwide and rank as the fifth most prevalent contributor to cancer-related deaths in women 71,72. In 2008, this neoplasm accounted for over 224,000 newly diagnosed cases and claimed approximately 140,000 lives globally, with a curability rate of less than 40% among affected women 73. Therefore, the development of drugs for the effective diagnosis or treatment of ovarian cancer is therefore a highly significant endeavor.

**Figure 5:**
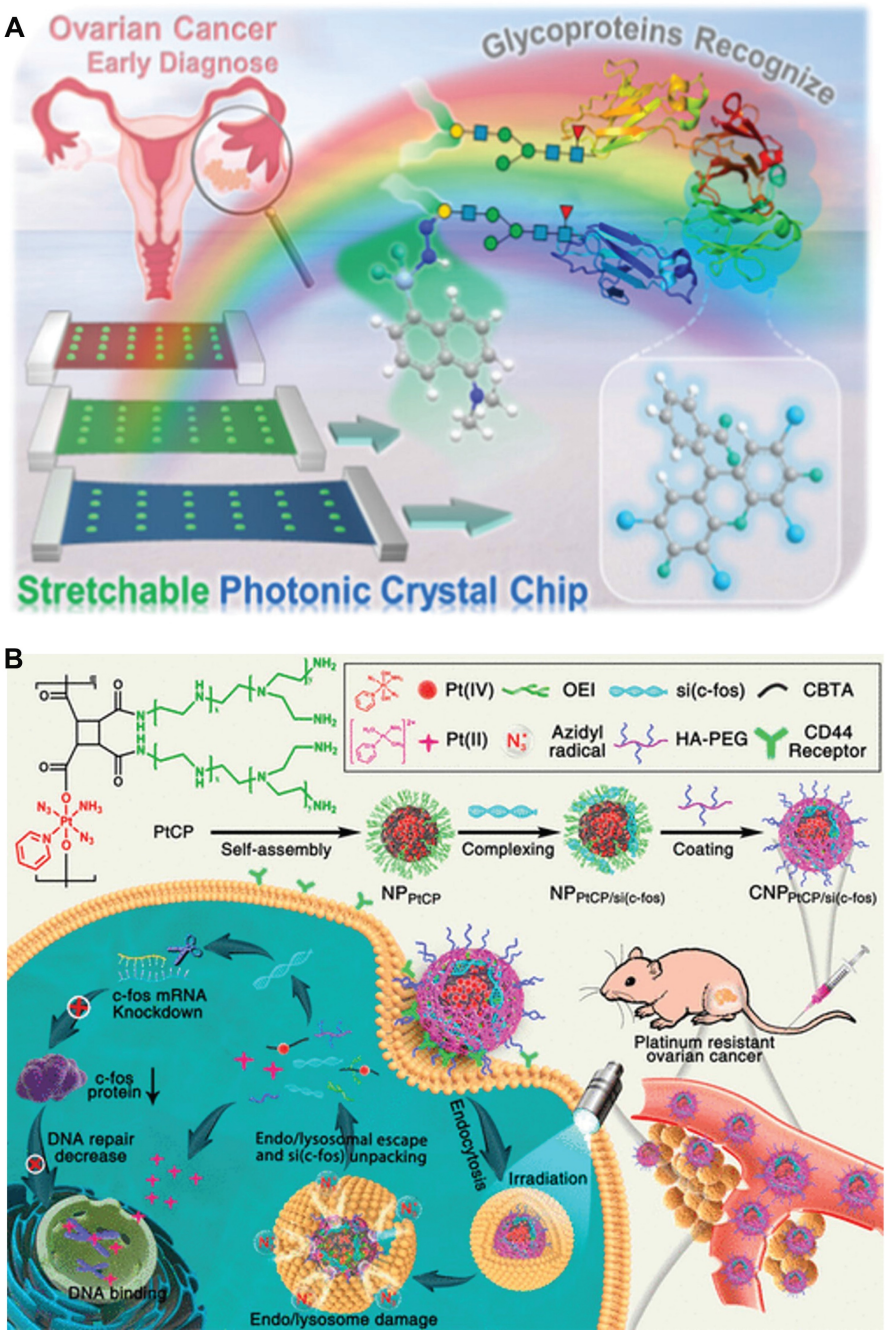
(A) Stretchable photoniccrystal-assisted glycoprotein identification for ovarian cancer diagnosis. (B) Schematic illustration of the photoactivatable Pt(IV) prodrug-backboned polymeric nanoparticle system for light-controlled efficient gene delivery and synergistic PACT and RNAi on platinum-resistant ovarian cancer. **(A)** Adapted with permission 75, copyright 2024, American Chemical Society.** (B)** Adapted with permission 76, copyright 2020, American Chemical Society.

As constituents of protein antigens, the glycoprotein index typically serves as an indicator of tumorigenesis and metastasis 74. Consequently, the detection of glycoproteins has been recognized as a therapeutic target or biomarker for ovarian cancer diagnosis, clinical treatment, and prognosis assessment. For example, Li* et al*. developed an approach for the early diagnosis of ovarian cancer by utilizing stretchable photonic crystals to efficiently identify glycoproteins in complex matrices (**Figure 5A**) 75. Specific methods are as follows: A double-indicator fluorescence sensor was prepared to independently respond to the protein stem and oligosaccharide segment of glycoproteins, thereby improving recognition accuracy. When tested on clinical samples from healthy subjects and patients with early, middle, and advanced ovarian cancer, this method successfully distinguished seven typical glycoproteins from proteins, sugars or mixed disruptors with 100% accuracy. In conclusion, this stretchable photonic crystal-assisted glycoprotein identification method provides an effective means for accurate and rapid diagnosis of ovarian cancer.

In addition to the diagnosis of ovarian cancer, nanotechnology can also be utilized for the treatment of ovarian cancer. For instance, Huang *et al*. developed a photoactivated Pt(IV) prodrug skeleton polymer nanoparticle system (CNP_PtCP/si(c-fos)_) for controlled small interfering RNA of c-fos (si(c-fos)) delivery and synergistic photoactivated chemotherapy (PACT) and RNA interference (RNAi) in Pt-resistant ovarian cancer (**Figure 5B**) 76. CNP_PtCP/si(c-fos)_ generated oxygen-independent N_3_• with low oxidation energy upon blue light irradiation at 430 nm, facilitating efficient escape from endonucleases/lysosomes through N_3_•-assisted photochemical internalization, thereby reducing gene inactivation. Subsequently, upon activation of the Pt(IV) prodrug, CNP_PtCP/si(c-fos)_ released active Pt(II), while simultaneously unpackaging si(c-fos). Both* in vitro* and *in vivo* experiments demonstrated that CNP_PtCP/si(c-fos)_ exhibited significant synergistic therapeutic efficacy against ovarian cancer with minimal toxicity. This pre-drug skeleton polymer nanoplatform for PACT hold great potential as a gene/drug co-delivery strategy for various challenging cancers.

### Regulation of the vaginal microbiome

The intricate community structure and dynamic nature of the vaginal microbiome play a pivotal role in female reproductive health. It exerts a central influence on various aspects, including fertility, pregnancy, prevention of pelvic inflammatory disease, as well as fungal, urinary, and sexually transmitted infections. Similar to other mucosal sites, the female reproductive tract harbors a distinct microbiome predominantly dominated by lactobacillus species that are indispensable for maintaining overall health and homeostasis. However, an imbalance in the vaginal microbiome disrupts this equilibrium and promotes the proliferation of pathogenic bacteria such as candida, leading to reproductive tract diseases. Therefore, exploring nanomedicine interventions holds significant implications for regulating the balance of the vaginal microbiome.

Wei* et al*. developed a nanozyme-probiotic responsive hydrogel-rGO@FeS_2_/Lactobacillus@HA with peroxide-like activity (POD) and the ability to regulate the vaginal microenvironment, effectively targeting *C. albicans* in candida vaginitis treatment while reducing disease recurrence 8. In this study, a rGO@FeS_2_ nanozyme-lactobacillus hyaluronic acid (HA) hydrogel for vaginal application was designed. At the infection site, pathogenic organisms such as *C. albicans* and bacteria secrete hyaluronidase to degrade HA, thereby releasing lactobacilli and rGO@FeS_2_ nanozyme. Lactobacillus produces lactic acid and H_2_O_2_ to lower vaginal pH, while rGO@FeS_2_ nanozyme exhibits POD-like activity under acidic conditions by catalyzing H_2_O_2_ into •OH for effective eradication of *C. albicans*, achieving simultaneous antifungal action and regulation of the vaginal microenvironment.

## Diseases of pregnancy

Preterm birth, defined as delivery occurring before 37 weeks of gestation, remains the leading cause of neonatal mortality and morbidity. Spontaneous preterm births, encompassing preterm labor and preterm premature rupture of membranes, are considered a syndrome resulting from various factors such as infection or inflammation, vascular disease, and uterine overdistension. Indicated preterm births commonly arise due to conditions like placental accreta spectrum, ectopic pregnancy, preeclampsia, and deep vein thrombosis during pregnancy. Therefore, enhancing the diagnosis and treatment strategies for these disorders holds paramount importance in preventing preterm birth.

### Pregastrulation period

#### Placenta accreta spectrum disorders

Placental accreta spectrum (PAS) disorders encompass invasive placenta, placenta accretion, and placenta penetration, wherein the placenta excessively infiltrates the myometrium during pregnancy and fails to detach during delivery 77. This can result in severe hemorrhage, occasionally leading to maternal mortality. Currently, several diagnostic techniques for PAS disease exist including serum analysis 78, ultrasound diagnosis 79, and magnetic resonance imaging 80; however, despite their effectiveness, these diagnostic methods lack sufficient accuracy to diagnose all cases of PAS. Moreover, their application is limited in areas with inadequate medical resources. Therefore, it is imperative to develop novel diagnostic techniques that enhance the precision of diagnosing PAS during pregnancy to ensure maternal safety.

**Figure 6:**
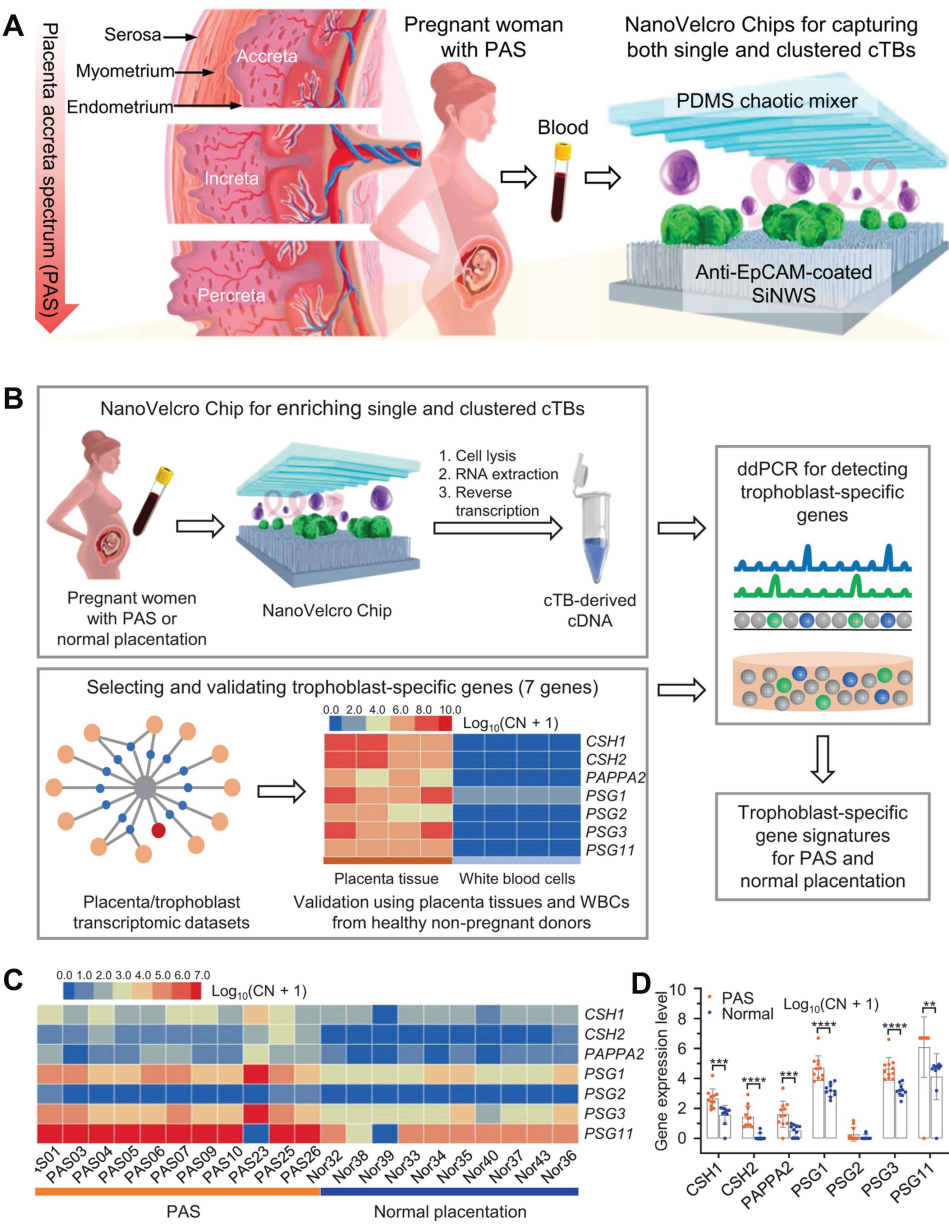
(A) NanoVelcro Chips for the detection of individual and clustered circulating trophoblasts in placenta accreta spectrum disorder. (B) Schematic exemplifying the overarching workflow for discerning trophoblast-specific genes in cTBs captured by NanoVelcro Chips. (C) Heat maps and (D) differences in gene expression depicting relative signal intensities of 7 trophoblast-specific genes in the cTBs. **(A-D)** Adapted with permission 81, copyright 2021, Springer Nature.

Zhu *et al*. developed an optimized NanoVelcro chip comprising ultra-thin silicon nanowires coated with antibodies capable of detecting circulating trophoblastic cells (cTBs), which constitute the placenta. This innovative technology enables the identification of placental cells associated with placental hyperplasia spectrum disorder in maternal blood samples during early pregnancy (**Figure 6A**) 81. Upon examination using the NanoVelcro chip, cTBs adhere to its surface and can be visualized under a microscope. Elevated counts or clusters of these cells in the bloodstream indicate an augmented risk of placental dysplasia. Utilizing the optimized chip, the researchers conducted an analysis on samples obtained from 168 pregnant women who were clinically diagnosed with either PAS, placenta previa, or normal placenta. Additionally, a control group consisting of 15 healthy non-pregnant females was included. The findings revealed a significantly higher presence of both individual and clustered cTB in participants with PAS compared to those without PAS. Single cTB was observed in the majority of pregnant women, with detection rates for PAS, placenta previa, and normal placenta being 98%, 85%, and 86% respectively. Notably, clustered cTB was found to be statistically more prevalent in cases of PAS than in both the placenta previa and normal placenta groups. Clustered cTB was identified in over 85% of PAS cases as opposed to only 20% for placenta previa and merely 14% for normal pregnancy. Subsequently, statistical analysis demonstrated that both single cTB and clustered cTB independently contributed towards predicting PAS. These findings were utilized to construct a logistic regression model which exhibited high accuracy when applied to independent sample sets. Overall results yielded a positive predictive value of approximately 84% along with a negative predictive value of 92% (**Figure 6B-D**). These findings suggested that NanoVelcro technology offered a promising approach for clinical diagnosis of PAS.

#### Ectopic pregnancy

Ectopic pregnancy (EP) is a potentially life-threatening condition in women where the fertilized egg cells fail to properly implant into the endometrium after traveling through the fallopian tubes 82. The majority (approximately 97%) of human ectopic implantation cases occur in the ampulla region of the fallopian tube; however, ectopic pregnancies can also develop in other locations, including the ovaries, cervix, abdomen, cesarean section scars, and different segments of the fallopian tube 83. Ectopic pregnancies represent a minority of all pregnancies, accounting for approximately 1%-2% of cases, but they have a significant impact on medical costs, with one study estimating an annual economic burden of nearly $1.1 billion. Bleeding in early pregnancy has historically been and continues to be a leading cause of maternal mortality in both developed and developing countries 84. Except in very rare cases of caesarean section pregnancies, ectopic implantation is not viable and poses serious risks, such as fallopian tube rupture, bleeding, hypovolemic shock, and potential fatality 85,86. Timely diagnosis and treatment are paramount in reducing the risks associated with fallopian tube rupture and subsequent bleeding.

**Figure 7:**
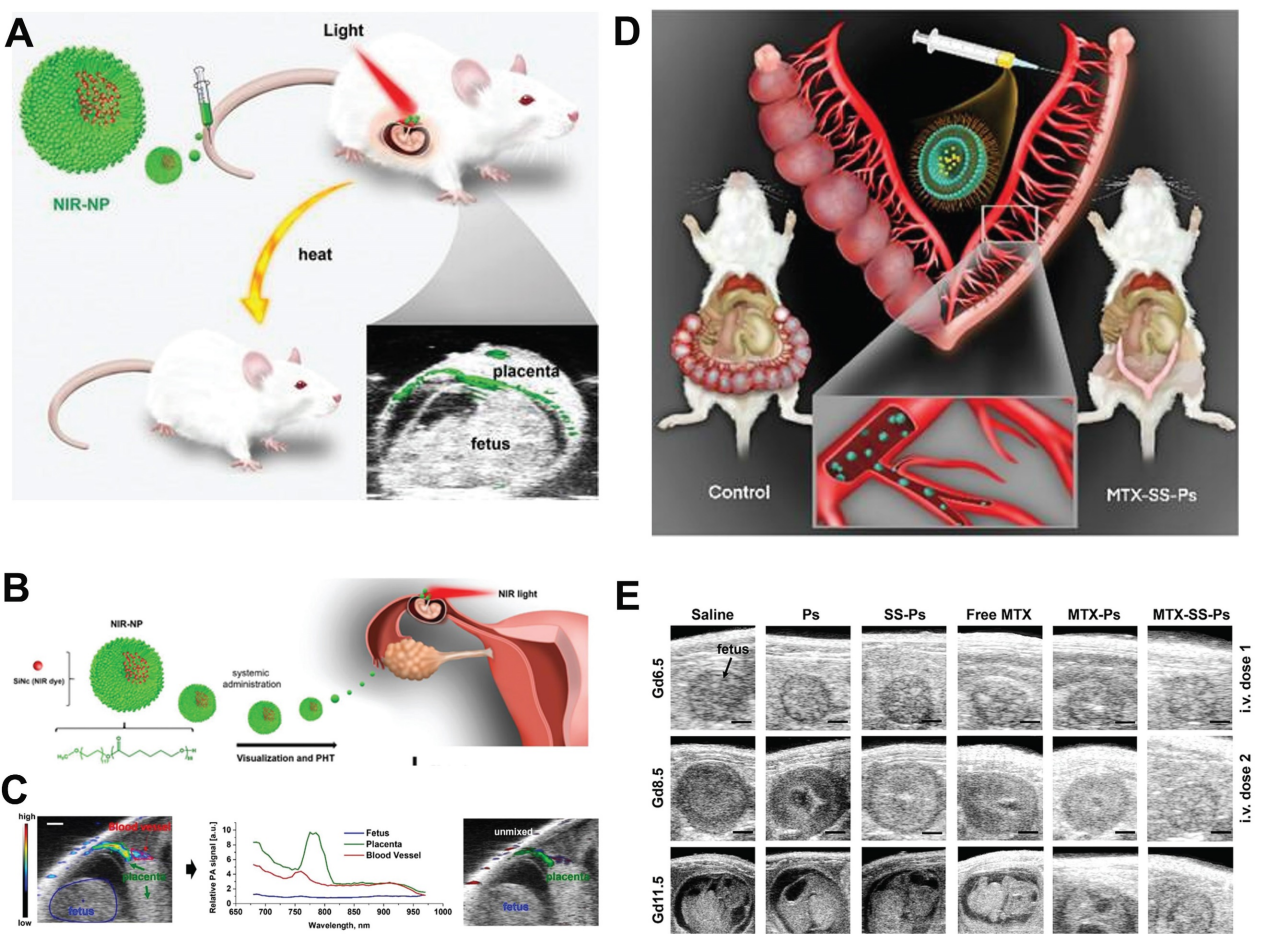
(A) Schematic elucidation of nanotechnology for placental visualization and its potential application in photothermal therapy for EP. (B) Schematic illustration of the light responsive nanodrug and their application in EP. (C) Photoacoustic imaging of EP. (D) Schematic illustration of GSH responsive nanodrug for management of EP. (E) US images of embryos from different group. **(A-C)** Adapted with permission 87, copyright 2023, Wiley-VCH. **(D, E)** Adapted with permission 85, Copyright 2023, Wiley-VCH.

Taratula *et al*. developed a NIR-NP nanomedicine for the diagnosis and treatment of EP, which comprised a near-infrared light-responsive molecule, silicon naphthyocyanine (SiNc), encapsulated in a biocompatible PEG-PCL nanoparticle (**Figure 7A, B**) 87. This NIR-NP exhibited a preferential accumulation in highly vascularized placentas. Upon systemic administration, the NIR-NP exhibited a targeted delivery of SiNc to the highly vascularized placental tissue rather than to the fetus. Subsequently, the utilization of fluorescence and photoacoustic imaging enabled the visualization of the developing placenta (**Figure 7C**). Following its accumulation in the placental tissue, this light-responsive NIR-NP also demonstrated the ability to generate heat when exposed to near-infrared light. This characteristic facilitated the application of selective, topical photohyperthermia, thus resulting in the disruption of placental function and subsequent pregnancy loss.

The initial treatment for EP, methotrexate (MTX) chemotherapy, exhibits a failure rate exceeding 10% and may result in severe complications or fatality. Inadequate accumulation of MTX at the site of ectopic implantation can contribute to treatment inefficacy. Stimuli-responsive drug delivery systems present an opportunity to address this predicament. For example, Taratula *et al*. designed a GSH responsive MTX polymersomes comprising an amphiphilic copolymer with disulfide bonds between PEG and PCL blocks. This design enhanced drug delivery efficiency and facilitated the cellular release of drug cargo within the placenta (**Figure 7D**) 85. Fluorescence and photoacoustic imaging analyses demonstrated that upon systemic administration, the developed polymersomes selectively accumulated at the implantation site in the first trimester of pregnant mice. Upon internalization into placental cells, high levels of intracellular GSH cleaved the disulfide bonds within the polymersomes, effectively releasing the MTX drug payload. Consequently, polymersomes-delivered MTX induced pregnancy termination, requiring a six-fold increase in dosage to achieve the same therapeutic outcome as free MTX under similar dosing regimens. Importantly, following pregnancy loss induced by MTX polymersomes, mice were able to conceive and successfully deliver healthy offspring (**Figure 7E**). This underscored the potential of the MTX-based nanomedicine in enhancing EP management, reducing associated mortality, and mitigating related costs.

### Mid-term pregnancy

#### Preeclampsia

Preeclampsia, a common cardiovascular complication of pregnancy that originates in the placenta and can result in various maternal and fetal complications, poses a significant threat to the survival of both mother and baby in severe cases 88. Typically occurring after 20 weeks of pregnancy, it is a leading cause of morbidity and mortality during pregnancy. Failure to diagnose this condition promptly may lead to serious complications such as eclampsia, premature birth, or even death. Therefore, the pressing necessity for the development of exquisitely sensitive point-of-care tests lies in their unparalleled capacity to evaluate the risk of progression towards preeclampsia and expedite prompt diagnosis, thereby empowering immediate clinical decision-making.

**Figure 8:**
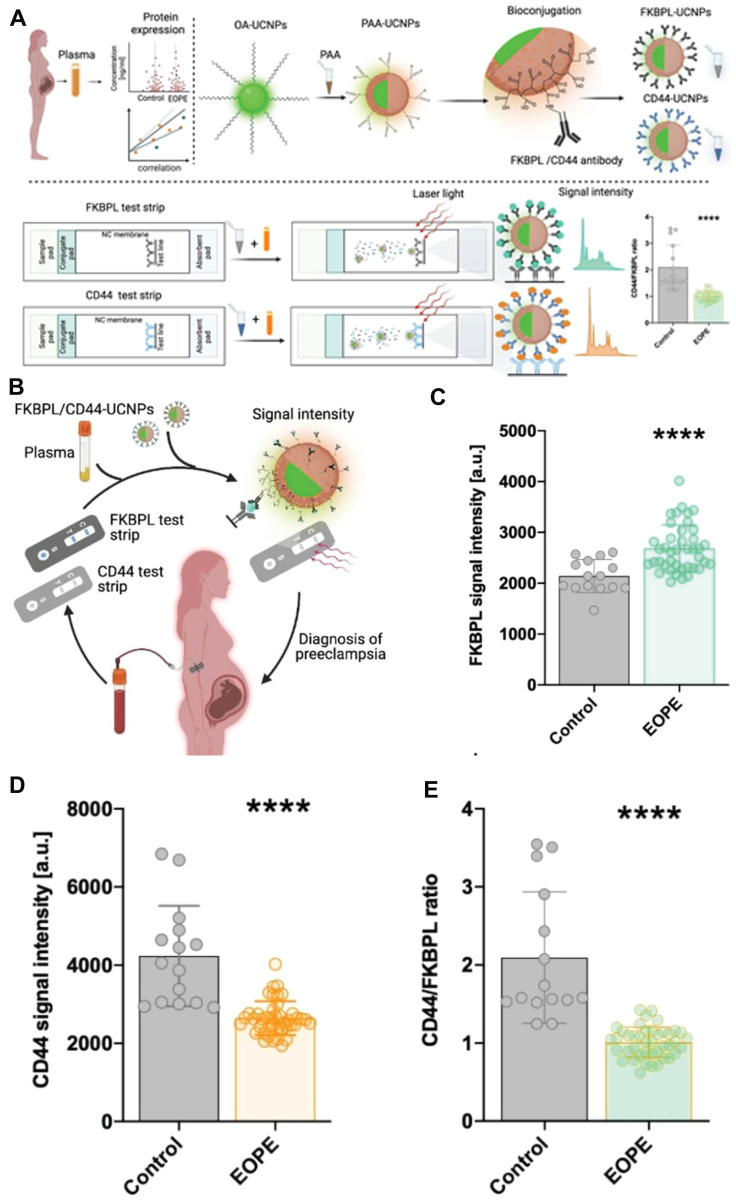
(A) Schematic illustration of the design principle of side-flow strip assay. (B) Schematic illustration of timely diagnosis of early-onset preeclampsia. (C) FKBPL signal intensity. (D) CD44 signal intensity. (E) CD44/FKBPL ratio. **(A-D)** Adapted with permission 90, copyright 2023, Wiley-VCH.

The biomarkers FKBPL and CD44 have been identified as potential indicators for predicting and diagnosing preeclampsia. By assessing the CD44/FKBPL ratio in plasma samples collected from pregnant women at the 20th week of gestation, it becomes feasible to evaluate the likelihood of developing preeclampsia 89. This approach facilitates the identification of pregnancies with a high risk that necessitate prompt diagnosis. Building upon this, McClements *et al*. developed a side-flow strip that incorporated up-conversion nanoparticles (UCNPs) and novel vascular biomarkers, such as FKBPL and CD44 proteins, in order to facilitate accurate and timely diagnosis of preeclampsia during pregnancy. This innovative approach has the potential to enhance the likelihood of preventing significant maternal complications and potential mortality (**Figure 8A**) 90. The bio-coupled UCNPs were mixed with varying concentrations of FKBPL and CD44 proteins in the running buffer for a duration of 10 minutes. Subsequently, the resulting mixture was applied to the sample pad, allowing it to migrate along the nitrocellulose membrane until protein binding occurs with the trapping antibody on the test line. The luminescence intensity of UCNPs on the test line was measured using a strip reader equipped with a 980 nm laser (**Figure 8A**). The findings unveiled a robust correlation between the signal intensity of UCNPs at 654 nm and the presence of FKBPL protein, showcasing an impressively lower detection threshold for FKBPL protein (approximately 10 pg mL^-1^), nearly fifteen times less than that achieved through ELISA. Similarly, the fluorescence emitted by UCNPs exhibited a positive association with CD44 concentration, resulting in an estimated detection limit of approximately 15 pg mL^-1^ - almost nine times superior to ELISA. Given the satisfactory performance exhibited by the developed side-flow strip in detecting FKBPL and CD44 biomarkers, the subsequent step involved verifying whether UCNPS-based side-flow strip can rapidly and reliably detect concentrations of FKBPL and CD44 in clinical samples. This particular type of test would prove advantageous for diagnosing preeclampsia at point-of-care settings. Based on the detection limits observed in side-flow strip, plasma concentrations were diluted through a mixture with a running buffer along with a FKBPL-UCNPs or CD44-UCNPs probe. The resulting UCNPS-plasma conjugate was then passed across the strip to measure fluorescence signals for FKBPL and CD44 respectively (**Figure 8C**). In comparison to the control group, patients with early-onset preeclampsia displayed a significantly elevated plasma FKBPL signal (approximately 20%) (**Figure 8D**), while preeclampsia samples demonstrated a significant decrease in CD44 signal strength (around 40%) (**Figure 8E**). Consequently, this indicates that the CD44/FKBPL intensity ratio is considerably lower in cases of early-onset preeclampsia when compared to the control group. The findings suggested that this side-flow strip had the potential to be utilized for the detection of various biomarkers in point-of-care settings, enabling early diagnosis of preeclampsia.

### Late pregnancy

#### Deep vein thrombosis in pregnancy

During pregnancy, deep vein thrombosis (DVT) can have severe consequences, including pulmonary embolism, recurrent miscarriages, restricted fetal growth, pre-eclampsia, and placental abruption. This condition poses a significant threat to both the mother and fetus, leading to complications, mortality, and financial burden. Pulmonary embolism, a common consequence of DVT, is the primary cause of maternal mortality. Clinical studies consistently show that DVT often occurs in the iliofemoral vein of the left lower limb during pregnancy due to hypercoagulation in later stages of gestation. This hypercoagulation disrupts normal blood clotting function, increasing the risk of clot formation. The pathogenesis of DVT involves elevated levels of procoagulant factors/proteins, endothelial cell damage, platelet activation, excessive production of reactive oxygen species (ROS), and inflammation. Symptoms of DVT, such as impaired blood flow and microthrombosis in the placenta, not only endanger maternal health but also impact fetal growth and development 91, 92. Therefore, it is crucial to implement medical interventions to reduce the incidence and progression of DVT during pregnancy.

**Figure 9:**
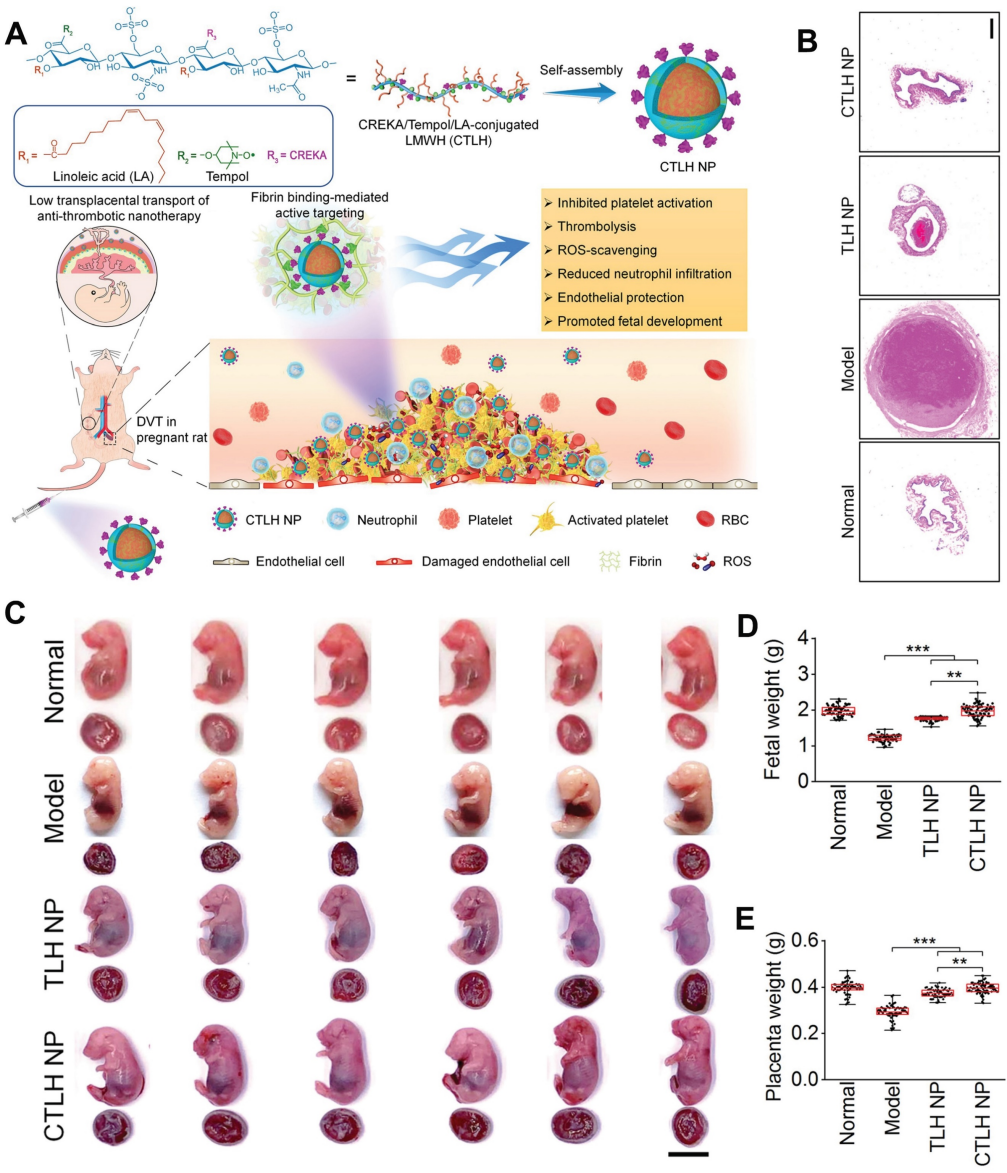
(A) Schematic representation of the engineering process for developing a multifunctional nanotherapy with antithrombotic properties, designed specifically for targeted treatment of deep vein thrombosis during pregnancy. (B) Histological sections of the left iliac veins stained with hematoxylin and eosin (H&E). (C) Digital images of representative rat fetuses and placentas obtained from pregnant rats with normal conditions or deep vein thrombosis. (D) Fetal and (E) placental weights of different groups. **(A-E)** Adapted with permission 93, copyright 2022, Springer Nature.

Zhang *et al*. developed a biologically active amphiphilic compound by covalently linking Tempol (antioxidative component) and linoleic acid (anti-inflammatory agent) to low molecular weight heparin (LMWH) derivative, resulting in the formation of multifunctional nanoparticles (TLH NP) 93. TLH NP exhibited promising efficacy in pregnant rats with DVT by specifically targeting and dissolving thrombi, alleviating vascular occlusion, and preventing thromboembolic recurrence. Consequently, this led to the amelioration of DVT-induced intrauterine growth restriction and fetal growth retardation. The therapeutic effects of TLH NP can be attributed to its ability to inhibit platelet aggregation, promote thrombolysis, mitigate local inflammation, reduce oxidative stress, facilitate endothelial repair, and enhance bioavailability. Furthermore, modification of the fibrin-binding peptide (CTLH NP) significantly enhanced the targeting efficiency and therapeutic efficacy of TLH NP. Notably, even at doses ten times higher than the tested therapeutic dose, the nanotherapies demonstrated no toxicity towards both maternal subjects and fetuses (**Figure 9A**). To assess the effectiveness of CTLH NP in pregnant rats with DVT, CTLH NP was found to eliminate iliac vein thrombosis more effectively and significantly reduce clot weight and length compared to other groups (**Figure 9B**). The presence of DVT can result in placental hypoxia, which indirectly impairs placental function through immunosuppression, delays trophoblast development, and induces trophoblast apoptosis. Furthermore, the hypoxic environment within placental trophoblast cells can lead to an upregulation of coagulation proteins and the deposition of microthrombi within placental blood vessels.

Collectively, these effects contribute to intrauterine fetal growth restriction. Therefore, the efficacy of l CTLH NP in reducing adverse pregnancy outcomes associated with DVT was assessed, the results suggested that CTLH NP exhibited a more pronounced efficacy in counteracting fetal and placental weight loss, reinstating structural integrity of the placenta, and fostering bone growth (**Figure 9C-E**). These results indicated that CTLH NP effectively prevented thrombus formation in the iliac vein of pregnant rats and reduces the risk of DVT by enhancing thrombus accumulation. Notably, the transplacental transfer of certain nanomaterials has been associated with pregnancy complications and neurotoxicity in mouse offspring. Nanoparticles with a diameter smaller than 80 nm and a cationic surface exhibit relatively high transport across the placental barrier, particularly during early stages of pregnancy when the placenta is not fully developed. To address this issue, the authors designed negatively charged LMWH nanotherapies with a size larger than 100 nm for administration in late-stage pregnancy, resulting in reduced transplacental penetration. Additionally, safety studies were conducted using a high dose (150 mg/kg, 10-fold higher than therapeutic doses) of nanotherapies administered to pregnant rats. Following treatment, no significant differences were observed in organ indices of major organs, fetuses, and placentas between control and nanotherapy groups; importantly, no placental abnormalities or fetal malformations were detected. The findings proposed an efficacious and secure approach to mitigate developmental disorders associated with DVT.

#### The application of nanomedicine in prenatal intervention for fetal health

Continuous advancements in DNA sequencing and prenatal diagnostic technology have facilitated the pre-birth diagnosis of numerous genetic disorders. Although certain diseases can be managed postnatally through protein or enzyme replacement therapy, irreversible damage may have already occurred after birth. Consequently, administering treatment in utero holds potential for enhanced efficacy due to the fetus's lower weight, enabling attainment of optimal therapeutic dosage, as well as the immaturity of the fetal immune system which may render it more receptive to alternative interventions. In terms of therapeutic protein replacement, mRNA distinguishes itself from other nucleic acids like DNA by not requiring nuclear entry and leveraging endogenous mechanisms to generate functional proteins. Theoretically capable of synthesizing a wide range of proteins, mRNA possesses the ability to transform cellular protein production into a veritable “drug factory”, thereby offering promising prospects for treating diverse ailments.

Riley* et al*. developed an intrauterine mRNA lipid nanoparticle (LNP) delivery system that has demonstrated favorable safety profiles and potential therapeutic effects in mouse models 94. This study laid the groundwork for future development of therapies for genetic disorders diagnosed prenatally. To identify possible non-viral delivery systems for therapeutic mRNAs, the researchers designed an LNP library. These particles, all less than 100 nm in size, can effectively entered the cells of the mouse fetus. The LNPs were composed of ionizable lipids, phospholipids, cholesterol, and lipid-anchored PEG, which were crucial for the structural integrity, stability, and intracellular mRNA delivery of the LNPs.

The researchers screened these LNPs to identify those capable of delivering functional mRNA to the fetal liver, lungs, and intestines, and accumulating in these organs. They also evaluated the maternal and fetal toxicity of these LNPs, finding them to be as safe or safer than benchmark preparations. After identifying LNPs that could accumulate in the fetal liver, lungs, and intestines with maximum efficiency and safety, the researchers employed easily traceable erythropoietin (EPO) mRNA to validate the therapeutic potential of these designs. The study showed that delivery of EPO mRNA into fetal hepatocytes increased the level of EPO protein in fetal blood circulation.

In conclusion, the researchers have developed and screened LNP platforms for efficient delivery of nucleic acids, which hold potential for addressing monogenic fetal diseases that currently lack adequate prenatal treatment options. These LNPs exhibited superior liver delivery, transfection efficiency, and safety profiles compared to commercially available delivery agents DLin-MC3-DMA and jetPEI, which are considered benchmarks for *in vivo* nucleic acid delivery. Although extensive evaluation has been conducted on the therapeutic potential of LNPs postnatally, limited research has been dedicated to assessing the utilization of ionizable LNPs prenataly. This study addresses this gap and establishes a foundation for the development and screening of nucleic acid delivery LNP platforms that could potentially be employed in the treatment of monogenic fetal diseases.

## Thorough assessments of nanodrug

The size, structure, aggregation, and administration methods of nanoparticles significantly influence the pharmacokinetics and safety profiles of nanomedicines, thereby determining their impact on maternal and infant health.

‌ 1. The impact of particle size: The particle size of nanodrug is a crucial determinant for its biological distribution and efficacy. Nanodrug typically exhibits a particle size below 100 nm, which introduces a pronounced scale effect compared to conventional pharmaceutical preparations. Upon transformation into nano preparations, the drug molecules undergo distinct action processes from their free counterparts, resulting in altered modes of action, enhanced action intensity, and modified *in vivo* transport mechanisms for both drug particles and biofilm within nano aggregates. This phenomenon can lead to significantly accelerated drug release from nanoparticles, thereby greatly improving drug solubility, bioavailability, and targeted delivery.

2. Structural effects: The interaction between nanomedicine and biological systems is influenced by its structural characteristics. For instance, cellular uptake of nanoparticles primarily occurs through phagocytosis and pinocytosis, while receptor-mediated endocytosis represents the predominant pathway for nanoparticle internalization. This process initiates upon ligand binding to specific receptors on the cell membrane, triggering a cascade of biochemical reactions that impact drug absorption and efficacy.

3. The influence of the degree of aggregation: The extent of aggregation of nanomedicine also plays a crucial role in determining its bioavailability and efficacy. The aggregation behavior of nanomedical drugs can significantly influence their distribution and metabolism within the body. Excessive aggregation may lead to reduced drug bioavailability, while appropriate levels of aggregation can facilitate targeted drug delivery and enhance therapeutic outcomes.

‌ 4. The route of administration should be carefully considered by pregnant women: In addition to conventional oral and intravenous administration, vaginal administration serves as a distinctive route of medication for pregnant women. The vaginal route of drug administration offers numerous advantages, such as bypassing hepatic first-pass metabolism and facilitating the use of lower drug dosages. Additionally, drugs administered vaginally can exploit the “uterine first-pass effect”, a phenomenon characterized by counter-current exchange between the venous, arterial, and lymphatic networks of the vagina and uterus. By harnessing this uterine first-pass effect, drugs can be selectively delivered to the upper reproductive tract without undergoing significant absorption or dilution into systemic circulation. Consequently, vaginal administration emerges as an appealing approach for women's health applications encompassing fertility assistance and prevention of preterm birth.

5. The impact of nanodrug on maternal and fetal health has dual implications: In the treatment of reproductive, obstetric, and gynecological diseases, concerns often arise regarding the potential effects of nanotherapy on gamete and embryo development as well as adverse impacts on female reproductive organs. Indeed, if not properly designed, nanomedicine may breach the placental barrier and directly affect the developing fetus. Research indicates that inhaled nanoparticles can traverse the placenta in pregnant rats during gestation, exerting long-term consequences on fetal health. However, through strategic design approaches such as functional nanoparticle modifications and targeted molecular conjugation, it is possible to selectively target diseased organs or tissues specific to pregnant women including placentas or fetuses. This approach reduces systemic drug therapy risks for fetuses by achieving comparable biological efficacy at lower blood drug concentrations while minimizing required dosages. Consequently, this mitigates potential adverse side effects faced by both mother and fetus alike. Moreover, advancements in current technology have facilitated the development of nanomedicine that can be readily transmitted into the fetal circulation or engineered to remain within the mother's system, thereby minimizing potential adverse effects on fetal growth and development while also reducing maternal dosage.

In summary, the fundamental principles and methodologies of nanodrug design primarily involve precise manipulation of substance structure and properties at the nanoscale to optimize and enhance their functionality. The size, structure, aggregation, and administration route of nanodrugs play a crucial role in their impact on maternal and fetal health, affecting not only therapeutic efficacy but also posing potential health risks. Therefore, careful consideration is necessary when utilizing nanodrugs.

## ‌Conclusions and outlook

Nanomaterials possess immense potential in the realm of women's reproductive health, as they have the capability to efficiently transport a wide range of therapeutic agents to specific sites within the reproductive system, thereby offering innovative treatment options for infertility, PCOS, and other related disorders. Moreover, nanomaterial-based imaging technologies hold the capacity to facilitate more precise and earlier detection of gynecological diseases, enabling timely intervention and improved disease management. Additionally, nanomaterials demonstrate significant promise in addressing pregnancy complications and enhancing maternal and child health. In this Review, we provide a comprehensive overview of recent advancements in nanomaterials for applications in regulating reproductive health in women (**Table 1**). This includes addressing preconception disorders such as PCOS, endometriosis, and gynecologic malignancy treatment; conditions during the pregastrulation period including PAS and EP; mid-term pregnancy ailments like preeclampsia; and late-stage pregnancy complications such as DVT during gestation, among others.

Reproductive health, the cornerstone of women's well-being, encompasses not only the nurturing ground for life but also the production hub of estrogen, a hormone crucial to women's beauty. The state of reproductive health is directly linked to women's beauty, youthfulness, and overall health. It goes beyond the absence of diseases and disorders in the reproductive process; it represents a holistic state of physical, psychological, and social well-being throughout this process. Given the unique nature of the female reproductive system, women face various health challenges related to fertility, menstruation, menopause, and more. Consequently, prioritizing women's reproductive health has become an imperative topic within both medical circles and society at large. However, current research on women's reproductive healthcare remains in its nascent stage with numerous issues and shortcomings. Therefore, it is essential to develop novel strategies for enhancing women's reproductive healthcare.

The emergence of nanomedicine presents a promising opportunity for the diagnosis and treatment of women's reproductive health. Despite significant advancements in the field of nanomedicine, its applications in regulating women's reproductive health are still at an early stage. Prior to clinical translation, nanomedicine in the regulation of women's reproductive health necessitates addressing the following challenges:

1. Improper utilization of nanomedicine can exert adverse effects on the reproductive system, such as compromising the development and maturation of oocytes within the ovaries, which are crucial for successful fertilization and conception in subsequent generations. However, upon entry into the human body, certain nanomaterials generate free radicals and oxides that infiltrate the ovary via blood circulation, thereby inducing toxic impacts on ovarian cells and oocytes. Consequently, this leads to aberrant egg morphology and diminished fertility. Therefore, it is imperative to establish a robust safety evaluation framework for nanomaterials to ensure their secure application.

2. The relationship between nanomedicine and the placental barrier remains elusive. Further research is required to elucidate how maternal administration can achieve the specific effect of nanomedicine on the placenta or fetus, ensuring safety for both mother and child, thereby facilitating early intervention and treatment of fetal dysplasia during pregnancy.

In summary, the design of nanomaterials has garnered increasing attention in the field of theranostics for women's reproductive health. As researchers deepen their understanding of the intricate relationship between nanomedicine and women's reproductive health, more promising applications of nanomaterials will continue to advance fundamental and clinical research in this domain.

## Figures and Tables

**Table 1:** A comprehensive overview of the application employed by nanosystems for regulating women's reproductive health

Disease type	Nanomaterial	Cargo	Application	References
Polycystic ovary syndrome (PCOS)	CHIT/CuNPs/Fe_3_O_4_ NPs/GrONPs/Au	Horseradish peroxidase-labeled SHBG antibody	specific detection of SHBG antigen in PCOS patients	49
PCOS	Nanocurcumin	Curcumin	Treatment of pancreatic function deficits associated with PCOS	57
Endometriosis	SiNc-NPs	Silyl phthalocyanine	Fluorescence imaging and photothermal treatment of endometriosis	66
Endometriosis	KDR-MN	Kinase insert domain receptor	Magnetothermal therapy of endometriosis	67
Cervical cancer	terrylenediimide (TDI) NPs	N/A	Photothermal treatment of cervical cancer	70
Ovarian cancer	CNP_PtCP/si(c-fos)_	Pt(IV) prodrug and si(c-fos)	Synergistic photoactivated chemotherapy and RNA interference on ovarian cancer	76
Vaginitis	Hydrogel-rGO@FeS_2_/Lactobacillus@HA	rGO@FeS_2_ nanozyme and lactobacillus	Simultaneously regulating the vaginal microenvironment and catalyzing the killing of *C. albicans*	8
Placental accreta spectrum (PAS)	NanoVelcro Chips	Anti-EpCAM-grafted silicon nanowire substrate	Capturing both single and clustered cTBs for for early detection of PAS disorder	81
Ectopic pregnancy (EP)	NIR-NP	Silicon naphthalocyanine	Photoacoustic imaging of the placenta and nanoparticle-mediated photo-hyperthermia for managing EP.	87
EP	MTX polymersomes	Methotrexate (MTX)	Management of EP.	85
Preeclampsia	Bio-coupled UCNPs	FKBPL or CD44 antibodies	Timely diagnosis of early-onset preeclampsia.	90
Deep vein thrombosis (DVT)	TLH NP	Tempol and linoleic acid	Effectively prevented thrombus formation in the iliac vein of pregnant rats and reduces the risk of DVT.	93
Fetal diseases	LNP	mRNA	Developing LNP platforms for nucleic acid delivery with applications in the treatment of monogenic fetal diseases.	94
